# Poly[diethyl­enetriammonium [aquadi-μ_2_-sulfato-sulfatolanthanum(III)]]

**DOI:** 10.1107/S1600536809022272

**Published:** 2009-06-17

**Authors:** Yuan-Rui Wang, Yong-Sheng Hu, Cui-Li Shi, Dan-Ping Li, Ya-Feng Li

**Affiliations:** a School of Chemical Engineering, Changchun University of Technology, Changchun 130012, People’s Republic of China

## Abstract

In the title compound, {(C_4_H_16_N_3_)[La(SO_4_)_3_(H_2_O)]}_*n*_, the La atom adopts an irregular LaO_9_ coordination geometry, including one bonded water mol­ecule. The three sulfate groups adopt both monodentate and bidentate coordination to the metal ions. Two of the sulfate groups serve as bridges in the (100) and (010) directions, yielding infinite sheets, whereas the third is pendant to one La^3+^ cation. The protonated organic species inter­acts with the layers by way of N—H⋯O hydrogen bonds, and O–H⋯O hydrogen bonds involving aqua ligands also occur.

## Related literature

For related lanthanide sulfate structures, see: Bataille & Louër (2004 ▶); Dan *et al.* (2004 ▶); Liu *et al.* (2005 ▶); Rao *et al.* (2006 ▶); Wickleder (2002 ▶); Xing *et al.* (2003 ▶).
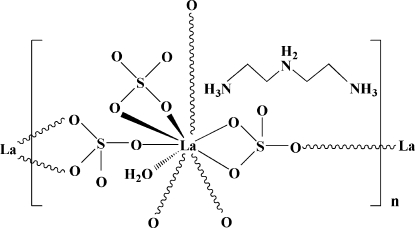


## Experimental

### 

#### Crystal data

(C_4_H_16_N_3_)[La(SO_4_)_3_(H_2_O)]
*M* *_r_* = 551.33
Monoclinic, 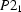

*a* = 6.7128 (13) Å
*b* = 10.442 (2) Å
*c* = 11.103 (2) Å
β = 93.94 (3)°
*V* = 776.4 (3) Å^3^
*Z* = 2
Mo *K*α radiation
μ = 3.23 mm^−1^
*T* = 293 K
0.45 × 0.31 × 0.06 mm

#### Data collection

Rigaku R-AXIS RAPID diffractometer
Absorption correction: multi-scan (*ABSCOR*; Higashi, 1995 ▶) *T*_min_ = 0.317, *T*_max_ = 0.830
7574 measured reflections
3429 independent reflections
3312 reflections with *I* > 2σ(*I*)
*R*_int_ = 0.028

#### Refinement

*R*[*F* ^2^ > 2σ(*F* ^2^)] = 0.021
*wR*(*F* ^2^) = 0.049
*S* = 1.17
3429 reflections
225 parameters
4 restraints
H atoms treated by a mixture of independent and constrained refinement
Δρ_max_ = 0.35 e Å^−3^
Δρ_min_ = −0.61 e Å^−3^
Absolute structure: Flack (1983 ▶), 1552 Friedel pairs
Flack parameter: −0.098 (11)

### 

Data collection: *PROCESS-AUTO* (Rigaku, 1998 ▶); cell refinement: *PROCESS-AUTO*; data reduction: *CrystalStructure* (Rigaku/MSC, 2002 ▶); program(s) used to solve structure: *SHELXS97* (Sheldrick, 2008 ▶); program(s) used to refine structure: *SHELXL97* (Sheldrick, 2008 ▶); molecular graphics: *DIAMOND* (Brandenburg, 2000 ▶); software used to prepare material for publication: *SHELXL97*.

## Supplementary Material

Crystal structure: contains datablocks I, global. DOI: 10.1107/S1600536809022272/hb2997sup1.cif

Structure factors: contains datablocks I. DOI: 10.1107/S1600536809022272/hb2997Isup2.hkl

Additional supplementary materials:  crystallographic information; 3D view; checkCIF report

## Figures and Tables

**Table 1:** Selected bond lengths (Å)

La1—O1*W*	2.445 (3)
La1—O1	2.474 (3)
La1—O7	2.475 (2)
La1—O5^i^	2.510 (3)
La1—O6	2.542 (3)
La1—O8^i^	2.577 (3)
La1—O3	2.580 (3)
La1—O9^ii^	2.583 (3)
La1—O2^ii^	2.615 (3)

Symmetry codes: (i) 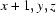; (ii) 
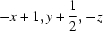
.

**Table 2:** Hydrogen-bond geometry (Å, °)

*D*—H⋯*A*	*D*—H	H⋯*A*	*D*⋯*A*	*D*—H⋯*A*
O1*W*—H1*F*⋯O4	0.841 (19)	1.98 (2)	2.775 (5)	157 (4)
O1*W*—H1*G*⋯O11^iii^	0.850 (18)	2.17 (4)	2.872 (5)	140 (4)
N1—H1*A*⋯O8^ii^	0.89	2.02	2.762 (5)	141
N1—H1*B*⋯O9^ii^	0.89	2.04	2.900 (5)	161
N1—H1*C*⋯O6^i^	0.89	2.07	2.874 (5)	150
N2—H2*B*⋯O11	0.90	1.96	2.798 (5)	155
N2—H2*A*⋯O2^iv^	0.90	2.18	3.015 (5)	154
N2—H2*A*⋯O4^iv^	0.90	2.28	2.981 (5)	134
N3—H3*A*⋯O5^v^	0.89	2.18	2.809 (5)	127
N3—H3*A*⋯O3^vi^	0.89	2.25	3.051 (5)	150
N3—H3*B*⋯O12^v^	0.89	1.95	2.834 (5)	174
N3—H3*C*⋯O10^vii^	0.89	2.08	2.784 (5)	135

Symmetry codes: (i) 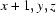; (ii) 
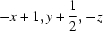
; (iii) 
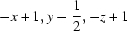
; (iv) 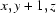; (v) 
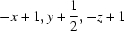
; (vi) 
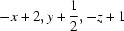
; (vii) 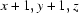.

**Fig. 1.**
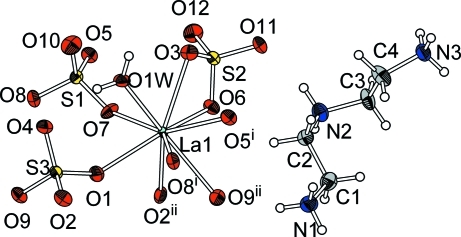
The asymmetric unit of (I), expanded to show the complete metal coordination and displacement ellipsoids at the 50% probability level. [Symmetry codes: (i) 1 + x, y, z; (ii) 1 - x, 1/2 + y, -z.]

**Fig. 2.**
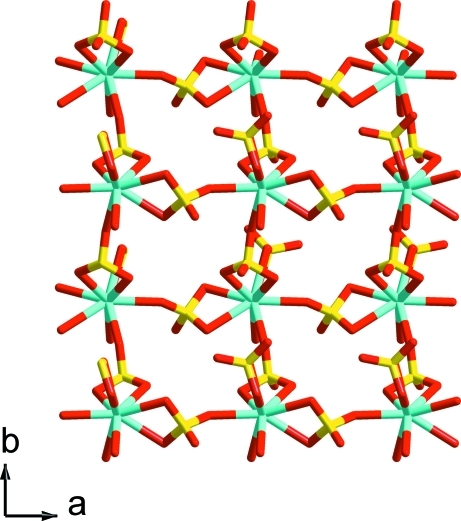
A stick plot of (I), displaying the layer paralleling ab planar formed by link the La with µ2-S(1)O4 and µ2-S(3)O4.

**Fig. 3.**
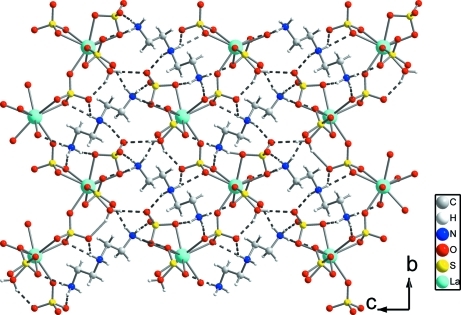
The ball-stick packing diagram of (I), viewed along (100) direction. The H-bond of N—H···O and OW—H···O hold together adjacent layer.

## supplementary crystallographic information

### Comment

Recently, a remarkable plenty of organically templated open-framework rare-earth metal sulfates have been obtained due to sulfate which gives the possibility of high framework dimensionalities and lanthanide with the high coordinated numbers (from 7-fold to 12-fold) according to larger ion diameters of rare-earth elements and in the sequel the complicated topologies (Wickleder, 2002; Rao, *et al.*, 2006). As SO_4_ group is able to adopt monodentate (S—O—Ln of -140°) and bidentate (S—O—Ln of -100°) to coordinate the lanthanide elements, the lanthanide sulfates are increasingly expanded with regard to the framework structures. Associated with reported two-dimensional lanthanide sulfates - [C_2_N_2_H_10_]^.^[Nd_2_(SO_4_)_4_](Dan, *et al.*, 2004), [C_2_N_2_H_10_]^.^[La_2_(H_2_O)_4_^.^(SO_4_)_4_]^.^2H_2_O (Xing, *et al.*, 2003), [C_6_H_14_N_2_]_2_^.^[La_2_(H_2_O)_4_^.^(SO_4_)_5_]^.^5H_2_O (Bataille, *et al.*, 2004), [C_2_N_2_H_10_]^.^[Nd_2_(H_2_O)_2_(SO_4_)_6_]^.^4H_2_O (Liu, *et al.*, 2005) and [C_6_N_2_H_14_]^.^[C_2_N_2_H_10_]^.^SO_4_^.^ [La_2_(H_2_O)_2_^.^(SO_4_)_6_]^.^4H_2_O (Dan, *et al.*, 2004), (I) keeps the distinct structure in which bridged µ_2_-SO_4_ afford one monodentate and one bidentate and grafted SO_4_ give the bidentate to the La cations.

The asymmetric unit of (I) comprises of twenty-four non-hydrogen atoms, 17 of which belong to the inorganic framework, including one La cation, three SO_4_ groups, one coordination water and one the organic template (four carbon atoms and three nitrogen atoms), as shown in Fig. 1. The two-dimensional layer of (I) is constructed from LaO_9_ and SO_4_ polyhedra. Three crystallographic independent S atoms, which are tetrahedrally coordinated by four O atoms with the S—O distances 1.458 (12) Å to 1.508 (4) Å, can be divided into two modes: S(1) and S(3) consist of three S—O—La linkages and links two La atoms through one bidentate and one monodentate; S(2) makes two S—O—La linkages as a ligand of one La atom through bidentate. The O—S—O angles are within the expected range for tetrahedral geometry. La ion is 9-coordinated by one monodentate and bidentate of µ_2_-S(1)O_4_ and µ_2_-S(3)O_4_, bidentate of S(2)O_4_ and one water molecule. The bond distances of La—O vary from 2.445 (4) to 2.617 (25) Å, whereas the angles of O—La—O are between 54.18 (10)° and 149.13 (10)°, which were found in other reported La compounds (Dan, *et al.*, 2004). The bond angles of S—O—La of bidentate coordination range from 99.27 (13)° to 101.15 (16)°, and the S—O—La of monodentate coordination is at 143.04 (13)° and 144.35 (19)°.

As shown in Fig.2, the layer of (I) is accomplished by connect the La by µ_2_-S(1)O_4_ along (100) direction and µ_2_-S(3)O_4_ along (010) direction. The S(2)O_4_ do not take part in the formation of layer and graft to the La ions by the bidentate coordination. The protonated H_3_DETA interact with the layer by the H-bond of N—H···O, which intergrate the Ow—H···O to hold togerther the adjacent layer to the supermolecular network (Fig.3).

### Experimental

La(NO_3_)_3_^.^6H_2_O (0.30 g, 0.7 mmol) was dissolved in 5 ml deionized water under stirring, and then H_2_SO_4_ (95%, 0.25 ml, 4.55 mmol) and DETA (0.33 ml, 4 mmol) were added drop-wise to a clear solution with pH = 4.0. After being continuously stirred for 3 h, the solution with the molar ratio of La(NO_3_)_3_^.^6H_2_O: 6.5H_2_SO_4_: 4.3DETA: 397H_2_O was transferred into a 23-ml autoclave and heated at 438 K for 5 days. After cooling to room temperature, colorless rods of (I) were collected by filtration as a single phase (yield 53% based on the La). The atomic ratio of La:S determined by EDX was 1:3, in consistence with the results of structural determination of (I).

### Refinement

Water H atoms were located in a difference Fourier map and were refined with O—H = 0.84 (2) Å, H···H = 1.37 (2) Å and *U*_iso_(H) = 1.2U_eq_(O). The remaining H-atoms were placed in calculated positions (C—H = 0.89 Å, N—H = 0.89–0.90 Å) and were included in the refinement as riding with U_iso_(H) = 1.2U_eq_(C, N).

### Crystal data

**Table:**

(C_4_H_16_N_3_)[La(SO_4_)_3_(H_2_O)]	*F*(000) = 544
*M**_r_* = 551.33	*D*_x_ = 2.358 Mg m^−^^3^
Monoclinic, *P*2_1_	Mo *K*α radiation, λ = 0.71073 Å
Hall symbol: P 2yb	Cell parameters from 2000 reflections
*a* = 6.7128 (13) Å	θ = 3.0–27.5°
*b* = 10.442 (2) Å	µ = 3.23 mm^−^^1^
*c* = 11.103 (2) Å	*T* = 293 K
β = 93.94 (3)°	Rod, colourless
*V* = 776.4 (3) Å^3^	0.45 × 0.31 × 0.06 mm
*Z* = 2	

### Data collection

**Table:**

Rigaku R-AXIS RAPID diffractometer	3429 independent reflections
Radiation source: fine-focus sealed tube	3312 reflections with *I* > 2σ(*I*)
graphite	*R*_int_ = 0.028
Detector resolution: 10.00 pixels mm^-1^	θ_max_ = 27.5°, θ_min_ = 3.0°
ω scans	*h* = −8→7
Absorption correction: multi-scan (*ABSCOR*; Higashi, 1995)	*k* = −13→13
*T*_min_ = 0.317, *T*_max_ = 0.830	*l* = −14→14
7574 measured reflections	

### Refinement

**Table:**

Refinement on *F*^2^	Secondary atom site location: difference Fourier map
Least-squares matrix: full	Hydrogen site location: inferred from neighbouring sites
*R*[*F*^2^ > 2σ(*F*^2^)] = 0.021	H atoms treated by a mixture of independent and constrained refinement
*wR*(*F*^2^) = 0.049	*w* = 1/[σ^2^(*F*_o_^2^) + (0.0089*P*)^2^] where *P* = (*F*_o_^2^ + 2*F*_c_^2^)/3
*S* = 1.17	(Δ/σ)_max_ = 0.006
3429 reflections	Δρ_max_ = 0.35 e Å^−^^3^
225 parameters	Δρ_min_ = −0.60 e Å^−^^3^
4 restraints	Absolute structure: Flack (1983), 1552 Friedel pairs
Primary atom site location: structure-invariant direct methods	Flack parameter: −0.098 (11)

### Special details

**Table:**

Geometry. All e.s.d.'s (except the e.s.d. in the dihedral angle between two l.s. planes) are estimated using the full covariance matrix. The cell e.s.d.'s are taken into account individually in the estimation of e.s.d.'s in distances, angles and torsion angles; correlations between e.s.d.'s in cell parameters are only used when they are defined by crystal symmetry. An approximate (isotropic) treatment of cell e.s.d.'s is used for estimating e.s.d.'s involving l.s. planes.
Refinement. Refinement of *F*^2^ against ALL reflections. The weighted *R*-factor *wR* and goodness of fit *S* are based on *F*^2^, conventional *R*-factors *R* are based on *F*, with *F* set to zero for negative *F*^2^. The threshold expression of *F*^2^ > σ(*F*^2^) is used only for calculating *R*-factors(gt) *etc*. and is not relevant to the choice of reflections for refinement. *R*-factors based on *F*^2^ are statistically about twice as large as those based on *F*, and *R*- factors based on ALL data will be even larger.

### Fractional atomic coordinates and isotropic or equivalent isotropic displacement parameters (Å^2^)

**Table:**

	*x*	*y*	*z*	*U*_iso_*/*U*_eq_	
La1	0.53138 (2)	0.36394 (2)	0.180431 (16)	0.00943 (6)	
S1	−0.00721 (14)	0.31013 (9)	0.24266 (10)	0.0156 (2)	
S2	0.42405 (15)	0.58541 (9)	0.36244 (10)	0.0176 (2)	
S3	0.42773 (14)	0.02938 (9)	0.06143 (10)	0.0167 (2)	
O1	0.4541 (4)	0.1705 (3)	0.0573 (3)	0.0236 (7)	
O2	0.6123 (4)	−0.0363 (3)	0.0235 (3)	0.0234 (7)	
O3	0.4904 (4)	0.4531 (3)	0.3940 (3)	0.0241 (7)	
O4	0.3862 (5)	−0.0119 (3)	0.1833 (3)	0.0287 (7)	
O5	−0.1328 (4)	0.4210 (3)	0.2793 (3)	0.0226 (6)	
O6	0.4374 (4)	0.5935 (3)	0.2274 (3)	0.0220 (7)	
O7	0.1623 (3)	0.3571 (4)	0.1758 (2)	0.0233 (6)	
O8	−0.1464 (4)	0.2337 (3)	0.1610 (3)	0.0217 (7)	
O9	0.2667 (4)	−0.0115 (3)	−0.0290 (3)	0.0226 (7)	
O10	0.0654 (5)	0.2351 (3)	0.3473 (3)	0.0333 (8)	
O11	0.5666 (5)	0.6790 (3)	0.4210 (3)	0.0285 (7)	
O12	0.2219 (4)	0.6107 (3)	0.3958 (3)	0.0320 (8)	
O1W	0.5151 (5)	0.1920 (3)	0.3288 (3)	0.0242 (7)	
H1F	0.478 (6)	0.119 (3)	0.303 (4)	0.029*	
H1G	0.554 (7)	0.188 (4)	0.403 (2)	0.029*	
N1	1.0903 (5)	0.6434 (3)	0.0681 (4)	0.0267 (8)	
H1A	1.1667	0.6620	0.0081	0.032*	
H1B	0.9960	0.5881	0.0422	0.032*	
H1C	1.1651	0.6089	0.1290	0.032*	
N2	0.7510 (5)	0.8376 (3)	0.2587 (4)	0.0270 (10)	
H2A	0.6714	0.8687	0.1970	0.032*	
H2B	0.6714	0.8081	0.3145	0.032*	
N3	1.0998 (5)	1.0100 (3)	0.4815 (4)	0.0265 (9)	
H3A	1.1893	0.9822	0.5384	0.032*	
H3B	0.9983	1.0464	0.5159	0.032*	
H3C	1.1567	1.0671	0.4352	0.032*	
C1	0.9941 (7)	0.7643 (4)	0.1105 (5)	0.0280 (10)	
H1D	0.9115	0.8028	0.0451	0.034*	
H1E	1.0952	0.8257	0.1387	0.034*	
C2	0.8674 (6)	0.7262 (4)	0.2127 (4)	0.0221 (9)	
H2C	0.7749	0.6595	0.1847	0.027*	
H2D	0.9532	0.6915	0.2786	0.027*	
C3	0.8728 (7)	0.9459 (4)	0.3132 (5)	0.0293 (11)	
H3D	0.9384	0.9900	0.2500	0.035*	
H3E	0.7849	1.0066	0.3492	0.035*	
C4	1.0257 (7)	0.9011 (4)	0.4067 (5)	0.0339 (12)	
H4A	0.9679	0.8373	0.4573	0.041*	
H4B	1.1359	0.8618	0.3682	0.041*	

### Atomic displacement parameters (Å^2^)

**Table:**

	*U*^11^	*U*^22^	*U*^33^	*U*^12^	*U*^13^	*U*^23^
La1	0.00845 (8)	0.00909 (8)	0.01059 (9)	−0.00017 (11)	−0.00037 (6)	−0.00017 (12)
S1	0.0125 (4)	0.0176 (4)	0.0165 (5)	−0.0001 (4)	−0.0004 (4)	0.0015 (4)
S2	0.0180 (5)	0.0177 (5)	0.0169 (5)	−0.0002 (4)	0.0002 (4)	−0.0024 (4)
S3	0.0182 (5)	0.0156 (4)	0.0161 (5)	−0.0013 (4)	0.0002 (4)	−0.0022 (4)
O1	0.0310 (17)	0.0163 (14)	0.0238 (18)	−0.0007 (13)	0.0034 (14)	−0.0025 (12)
O2	0.0223 (16)	0.0224 (14)	0.0253 (18)	0.0088 (12)	0.0006 (13)	−0.0037 (13)
O3	0.0279 (17)	0.0219 (14)	0.0226 (18)	0.0050 (13)	0.0023 (14)	0.0011 (13)
O4	0.0431 (19)	0.0273 (16)	0.0161 (18)	−0.0080 (15)	0.0038 (14)	−0.0004 (13)
O5	0.0148 (14)	0.0283 (14)	0.0247 (17)	0.0027 (13)	0.0012 (13)	−0.0069 (13)
O6	0.0255 (16)	0.0225 (14)	0.0175 (17)	0.0009 (13)	−0.0028 (13)	−0.0019 (12)
O7	0.0146 (11)	0.0317 (14)	0.0243 (14)	0.0013 (18)	0.0053 (10)	0.010 (2)
O8	0.0191 (14)	0.0188 (14)	0.0268 (18)	−0.0017 (12)	−0.0004 (13)	−0.0053 (12)
O9	0.0210 (15)	0.0258 (15)	0.0204 (18)	0.0016 (13)	−0.0035 (12)	−0.0051 (13)
O10	0.0318 (18)	0.0370 (18)	0.030 (2)	0.0032 (16)	−0.0058 (15)	0.0145 (15)
O11	0.0305 (17)	0.0313 (16)	0.0228 (18)	−0.0111 (14)	−0.0037 (14)	−0.0056 (14)
O12	0.0224 (15)	0.0356 (17)	0.039 (2)	0.0066 (14)	0.0108 (15)	−0.0046 (16)
O1W	0.0368 (17)	0.0208 (15)	0.0153 (17)	−0.0009 (15)	0.0034 (14)	0.0021 (13)
N1	0.0235 (19)	0.033 (2)	0.024 (2)	−0.0013 (17)	0.0031 (16)	0.0009 (17)
N2	0.0222 (16)	0.027 (3)	0.031 (2)	0.0017 (15)	−0.0033 (15)	−0.0011 (16)
N3	0.030 (2)	0.0243 (18)	0.024 (2)	−0.0041 (17)	−0.0034 (17)	0.0005 (16)
C1	0.028 (2)	0.026 (2)	0.030 (3)	−0.0006 (19)	0.000 (2)	0.004 (2)
C2	0.023 (2)	0.020 (2)	0.024 (2)	0.0011 (17)	0.0016 (18)	0.0003 (18)
C3	0.035 (3)	0.0159 (19)	0.036 (3)	−0.0007 (19)	−0.008 (2)	−0.0034 (19)
C4	0.050 (3)	0.022 (2)	0.028 (3)	0.007 (2)	−0.011 (2)	−0.0010 (18)

### Geometric parameters (Å, °)

**Table:**

La1—O1W	2.445 (3)	N1—C1	1.508 (6)
La1—O1	2.474 (3)	N1—H1A	0.8900
La1—O7	2.475 (2)	N1—H1B	0.8900
La1—O5^i^	2.510 (3)	N1—H1C	0.8900
La1—O6	2.542 (3)	N2—C3	1.498 (5)
La1—O8^i^	2.577 (3)	N2—C2	1.510 (5)
La1—O3	2.580 (3)	N2—H2A	0.9000
La1—O9^ii^	2.583 (3)	N2—H2B	0.9000
La1—O2^ii^	2.615 (3)	N3—C4	1.474 (5)
S1—O10	1.457 (3)	N3—H3A	0.8900
S1—O7	1.484 (3)	N3—H3B	0.8900
S1—O8	1.488 (3)	N3—H3C	0.8900
S1—O5	1.504 (3)	C1—C2	1.517 (6)
S2—O12	1.455 (3)	C1—H1D	0.9700
S2—O11	1.486 (3)	C1—H1E	0.9700
S2—O3	1.486 (3)	C2—H2C	0.9700
S2—O6	1.511 (3)	C2—H2D	0.9700
S3—O4	1.465 (3)	C3—C4	1.485 (6)
S3—O1	1.485 (3)	C3—H3D	0.9700
S3—O9	1.486 (3)	C3—H3E	0.9700
S3—O2	1.501 (3)	C4—H4A	0.9700
O1W—H1F	0.841 (19)	C4—H4B	0.9700
O1W—H1G	0.850 (18)		
			
O1W—La1—O1	75.84 (11)	S3—O1—La1	144.5 (2)
O1W—La1—O7	84.36 (11)	S3—O2—La1^iii^	99.36 (14)
O1—La1—O7	78.07 (11)	S2—O3—La1	99.67 (16)
O1W—La1—O5^i^	87.70 (11)	S1—O5—La1^iv^	101.69 (14)
O1—La1—O5^i^	125.67 (9)	S2—O6—La1	100.57 (14)
O7—La1—O5^i^	152.06 (10)	S1—O7—La1	143.12 (17)
O1W—La1—O6	122.10 (10)	S1—O8—La1^iv^	99.29 (14)
O1—La1—O6	146.90 (10)	S3—O9—La1^iii^	101.23 (14)
O7—La1—O6	76.67 (11)	La1—O1W—H1F	117 (3)
O5^i^—La1—O6	85.11 (9)	La1—O1W—H1G	132 (3)
O1W—La1—O8^i^	75.26 (11)	H1F—O1W—H1G	110 (3)
O1—La1—O8^i^	70.62 (9)	C1—N1—H1A	109.5
O7—La1—O8^i^	145.87 (10)	C1—N1—H1B	109.5
O5^i^—La1—O8^i^	55.10 (9)	H1A—N1—H1B	109.5
O6—La1—O8^i^	137.43 (9)	C1—N1—H1C	109.5
O1W—La1—O3	68.48 (10)	H1A—N1—H1C	109.5
O1—La1—O3	140.45 (10)	H1B—N1—H1C	109.5
O7—La1—O3	81.93 (10)	C3—N2—C2	115.9 (3)
O5^i^—La1—O3	70.24 (10)	C3—N2—H2A	108.3
O6—La1—O3	55.07 (9)	C2—N2—H2A	108.3
O8^i^—La1—O3	114.26 (10)	C3—N2—H2B	108.3
O1W—La1—O9^ii^	149.15 (10)	C2—N2—H2B	108.3
O1—La1—O9^ii^	98.68 (10)	H2A—N2—H2B	107.4
O7—La1—O9^ii^	124.77 (10)	C4—N3—H3A	109.5
O5^i^—La1—O9^ii^	70.70 (10)	C4—N3—H3B	109.5
O6—La1—O9^ii^	78.89 (10)	H3A—N3—H3B	109.5
O8^i^—La1—O9^ii^	74.33 (10)	C4—N3—H3C	109.5
O3—La1—O9^ii^	120.71 (10)	H3A—N3—H3C	109.5
O1W—La1—O2^ii^	147.69 (10)	H3B—N3—H3C	109.5
O1—La1—O2^ii^	78.24 (10)	N1—C1—C2	106.8 (4)
O7—La1—O2^ii^	71.61 (9)	N1—C1—H1D	110.4
O5^i^—La1—O2^ii^	123.41 (9)	C2—C1—H1D	110.4
O6—La1—O2^ii^	73.73 (10)	N1—C1—H1E	110.4
O8^i^—La1—O2^ii^	113.54 (10)	C2—C1—H1E	110.4
O3—La1—O2^ii^	126.54 (10)	H1D—C1—H1E	108.6
O9^ii^—La1—O2^ii^	54.16 (9)	N2—C2—C1	112.4 (3)
O10—S1—O7	110.52 (18)	N2—C2—H2C	109.1
O10—S1—O8	111.06 (19)	C1—C2—H2C	109.1
O7—S1—O8	110.06 (18)	N2—C2—H2D	109.1
O10—S1—O5	111.2 (2)	C1—C2—H2D	109.1
O7—S1—O5	110.1 (2)	H2C—C2—H2D	107.9
O8—S1—O5	103.72 (16)	C4—C3—N2	112.1 (3)
O12—S2—O11	110.7 (2)	C4—C3—H3D	109.2
O12—S2—O3	112.23 (19)	N2—C3—H3D	109.2
O11—S2—O3	109.55 (19)	C4—C3—H3E	109.2
O12—S2—O6	111.26 (19)	N2—C3—H3E	109.2
O11—S2—O6	108.49 (18)	H3D—C3—H3E	107.9
O3—S2—O6	104.40 (17)	N3—C4—C3	109.9 (4)
O4—S3—O1	110.57 (18)	N3—C4—H4A	109.7
O4—S3—O9	111.30 (18)	C3—C4—H4A	109.7
O1—S3—O9	110.28 (18)	N3—C4—H4B	109.7
O4—S3—O2	109.77 (19)	C3—C4—H4B	109.7
O1—S3—O2	109.99 (18)	H4A—C4—H4B	108.2
O9—S3—O2	104.78 (17)		
			
O4—S3—O1—La1	20.4 (4)	O11—S2—O6—La1	121.73 (16)
O4—S3—O1—La1	20.4 (4)	O3—S2—O6—La1	4.98 (17)
O9—S3—O1—La1	144.0 (3)	O1W—La1—O6—S2	11.55 (19)
O2—S3—O1—La1	−101.0 (3)	O1—La1—O6—S2	127.22 (17)
La1^iii^—S3—O1—La1	−159.2 (2)	O7—La1—O6—S2	86.02 (15)
O1W—La1—O1—S3	−4.8 (3)	O5^i^—La1—O6—S2	−72.65 (15)
O7—La1—O1—S3	−91.9 (3)	O8^i^—La1—O6—S2	−92.26 (18)
O5^i^—La1—O1—S3	71.8 (3)	O3—La1—O6—S2	−3.38 (12)
O6—La1—O1—S3	−132.8 (3)	O9^ii^—La1—O6—S2	−143.91 (15)
O8^i^—La1—O1—S3	74.3 (3)	O2^ii^—La1—O6—S2	160.44 (16)
O3—La1—O1—S3	−30.7 (4)	S1^i^—La1—O6—S2	−79.21 (14)
O9^ii^—La1—O1—S3	144.2 (3)	S3^ii^—La1—O6—S2	−170.95 (15)
O2^ii^—La1—O1—S3	−165.3 (3)	O10—S1—O7—La1	−4.0 (4)
S1^i^—La1—O1—S3	71.8 (3)	O8—S1—O7—La1	−127.0 (3)
S2—La1—O1—S3	−76.5 (4)	O5—S1—O7—La1	119.3 (4)
S3^ii^—La1—O1—S3	169.9 (3)	La1^iv^—S1—O7—La1	174.6 (2)
O4—S3—O2—La1^iii^	125.92 (15)	O1W—La1—O7—S1	16.6 (4)
O4—S3—O2—La1^iii^	125.92 (15)	O1—La1—O7—S1	93.2 (4)
O1—S3—O2—La1^iii^	−112.21 (16)	O5^i^—La1—O7—S1	−57.7 (5)
O9—S3—O2—La1^iii^	6.31 (17)	O6—La1—O7—S1	−108.3 (4)
O12—S2—O3—La1	115.73 (19)	O8^i^—La1—O7—S1	69.6 (4)
O11—S2—O3—La1	−120.90 (17)	O3—La1—O7—S1	−52.4 (4)
O11—S2—O3—La1	−120.90 (17)	O9^ii^—La1—O7—S1	−174.4 (3)
O6—S2—O3—La1	−4.89 (17)	O2^ii^—La1—O7—S1	174.7 (4)
O1W—La1—O3—S2	−163.00 (19)	S1^i^—La1—O7—S1	15.3 (6)
O1—La1—O3—S2	−135.94 (15)	S2—La1—O7—S1	−79.7 (4)
O7—La1—O3—S2	−75.91 (16)	S3^ii^—La1—O7—S1	−178.3 (4)
O5^i^—La1—O3—S2	101.50 (16)	O10—S1—O8—La1^iv^	115.43 (17)
O6—La1—O3—S2	3.43 (12)	O7—S1—O8—La1^iv^	−121.86 (17)
O8^i^—La1—O3—S2	135.54 (13)	O5—S1—O8—La1^iv^	−4.05 (18)
O9^ii^—La1—O3—S2	49.94 (18)	O4—S3—O9—La1^iii^	−125.00 (16)
O2^ii^—La1—O3—S2	−16.0 (2)	O4—S3—O9—La1^iii^	−125.00 (16)
S1^i^—La1—O3—S2	119.34 (14)	O1—S3—O9—La1^iii^	111.89 (16)
S3^ii^—La1—O3—S2	18.23 (18)	O2—S3—O9—La1^iii^	−6.43 (18)
O1—S3—O4—O4	0.00 (18)	O12—S2—O11—O11	0.0 (3)
O9—S3—O4—O4	0.00 (10)	O3—S2—O11—O11	0.0 (3)
O2—S3—O4—O4	0.00 (13)	O6—S2—O11—O11	0.0 (4)
La1^iii^—S3—O4—O4	0.00 (5)	La1—S2—O11—O11	0.0 (4)
O10—S1—O5—La1^iv^	−115.22 (18)	C3—N2—C2—C1	−62.8 (5)
O7—S1—O5—La1^iv^	121.94 (14)	N1—C1—C2—N2	−176.3 (3)
O8—S1—O5—La1^iv^	4.19 (18)	C2—N2—C3—C4	−51.4 (6)
O12—S2—O6—La1	−116.29 (17)	N2—C3—C4—N3	−163.4 (4)
O11—S2—O6—La1	121.73 (16)		

Symmetry codes: (i) *x*+1, *y*, *z*; (ii) −*x*+1, *y*+1/2, −*z*; (iii) −*x*+1, *y*−1/2, −*z*; (iv) *x*−1, *y*, *z*.

### Hydrogen-bond geometry (Å, °)

**Table:**

*D*—H···*A*	*D*—H	H···*A*	*D*···*A*	*D*—H···*A*
O1W—H1F···O4	0.84 (2)	1.98 (2)	2.775 (5)	157 (4)
O1W—H1G···O11^v^	0.85 (2)	2.17 (4)	2.872 (5)	140 (4)
N1—H1A···O8^ii^	0.89	2.02	2.762 (5)	141
N1—H1B···O9^ii^	0.89	2.04	2.900 (5)	161
N1—H1C···O6^i^	0.89	2.07	2.874 (5)	150
N2—H2B···O11	0.90	1.96	2.798 (5)	155
N2—H2A···O2^vi^	0.90	2.18	3.015 (5)	154
N2—H2A···O4^vi^	0.90	2.28	2.981 (5)	134
N3—H3A···O5^vii^	0.89	2.18	2.809 (5)	127
N3—H3A···O3^viii^	0.89	2.25	3.051 (5)	150
N3—H3B···O12^vii^	0.89	1.95	2.834 (5)	174
N3—H3C···O10^ix^	0.89	2.08	2.784 (5)	135

Symmetry codes:  (v) −*x*+1, *y*−1/2, −*z*+1; (ii) −*x*+1, *y*+1/2, −*z*; (i) *x*+1, *y*, *z*; (vi) *x*, *y*+1, *z*; (vii) −*x*+1, *y*+1/2, −*z*+1; (viii) −*x*+2, *y*+1/2, −*z*+1; (ix) *x*+1, *y*+1, *z*.
